# CXCR1/2 pathways in paclitaxel-induced neuropathic pain

**DOI:** 10.18632/oncotarget.15533

**Published:** 2017-02-20

**Authors:** Brandolini Laura, Benedetti Elisabetta, Ruffini Pier Adelchi, Russo Roberto, Cristiano Loredana, Antonosante Andrea, d'Angelo Michele, Castelli Vanessa, Giordano Antonio, Allegretti Marcello, Cimini Annamaria

**Affiliations:** ^1^ Dompé Farmaceutici SpA, Via Campo di Pile, L'Aquila, Italy; ^2^ Department of Life, Health and Environmental Sciences, University of L'Aquila, Italy; ^3^ Dompé Farmaceutici SpA, Via Santa Lucia, Milano, Italy; ^4^ Department of Pharmacy, University of Naples Federico II, Italy; ^5^ Department of Medicine, Surgery and Neuroscience, University of Siena, Siena, Italy; ^6^ Sbarro Institute for Cancer Research and Molecular Medicine and Center for Biotechnology, Temple University, Philadelphia, USA; ^7^ National Institute for Nuclear Physics (INFN), Gran Sasso National Laboratory (LNGS), Assergi, Italy

**Keywords:** chronic pain, chemotherapy, microtubules, IL-8, CXCR1/2 inhibitor

## Abstract

Chemotherapy-induced peripheral neuropathy (CIPN) is a type of neuropathic pain that represents a frequent and serious consequence of chemotherapy agents. Over the last years, significant progress has been achieved in elucidating the underlying pathogenesis of CIPN. The interference of taxanes with microtubule has been proposed as a mechanism that leads to altered axonal transport and to permanent neurological damages. The inflammatory process activated by chemotherapeutic agents has been considered as a potential trigger of nociceptive process in CIPN.

In this study we investigated the effect of reparixin, an inhibitor of CXCR1/CXCR2, in suppressing the development of paclitaxel-induced nociception in rats. Moreover, reparixin activity in reversing the neurotoxic effects induced by paclitaxel or GRO/KC in F11 cells was also analyzed.

Reparixin administered by continuous infusion ameliorated paclitaxel-induced mechanical and cold allodynia in rats. In F11 cells, reparixin was able to inhibit the increase of acetyladed α-tubulin induced both by paclitaxel and GRO/KC. The subsequent experiments were performed in order to dissect the signal transduction pathways under GRO/KC control, eventually modulated by paclitaxel and/or reparixin. To this aim we found that reparixin significantly counteracted p-FAK, p-JAK2/p-STAT3, and PI3K-p-cortactin activation induced either by paclitaxel or GRO/KC.

Overall the present results have identified IL-8/CXCR1/2 pathway as a mechanism involved in paclitaxel-induced peripheral neuropathy. In particular, the obtained data suggest that the inhibition of CXCR1/2 combined with standard taxane therapy, in addition to potentiating the taxane anti-tumor activity can reduce chemotherapy-induced neurotoxicity, thus giving some insight for the development of novel treatments.

## INTRODUCTION

Chemotherapy-induced peripheral neuropathy (CIPN) is a common and potentially disabling side effect of many anticancer drugs [1]. The most frequent symptoms are pain, numbness, or tingling in the hands or feet [1–2]. In a recent meta-analysis of several studies involving 4179 patients on various chemotherapeutic regimens, the prevalence of CIPN was 68.1% within the first month of treatment, 60.0% at 3 months, and 30.0% at 6 months [3]. With such a high prevalence of disease, CIPN has important clinical implications significantly impacting the quality of life and often resulting in treatment discontinuation, which may ultimately affect overall survival [4, 2]. Despite the intensive research dedicated to the identification of therapeutic options able to prevent or treat CIPN [5], the medical need remains very high.

The pathogenesis of CIPN has not been fully elucidated but it is noteworthy that the overall neuropathy symptom profile appears to be substantially shared across different classes of chemotherapeutic agents including taxanes, platinum, proteasome inhibitors, and vinca-alkaloids. Peripheral nerve degeneration or small fiber neuropathy is generally accepted as the underlying mechanism in the development of CIPN [6–7] but several studies point out that neuropathic pain caused by anticancer agents may occur early after the first infusion in the absence of damage to intra-epidermal nerve fibers or axonal degeneration in peripheral nerves [8–9].

Microtubule-stabilizing agents (MTSAs), including taxanes, are effective chemotherapeutic agents for the treatment of many oncologic conditions [10]. Although it is clear that chemotherapeutic agents that damage microtubules, interrupt mitochondrial function, or directly target DNA may affect the integrity and functionality of axons thus leading to peripheral nerve degeneration, several preclinical and clinical evidences concur to suggest a common physio-pathological mechanism partially independent from the antineoplastic drug molecular target.

The inflammatory process activated by chemotherapeutic agents has been considered a potential trigger of the nociceptive process in CIPN [11–12] and the chemotherapy-driven release of proinflammatory and chemotactic cytokines (chemokines) has been identified as one of the primary mechanisms regulating the neuro-immune communication.

Chemotherapeutic exposure consistently induces production and release of pro-inflammatory cytokines such as TNF-α, IL-1β, IL-6 and chemokines such as IL-8 and MCP-1 [13–15].

Pro-inflammatory cytokines can contribute to neural damage not only by activating the inflammatory process but also by a direct receptor-mediated activity on neurons and glial cells [16–20]. Among the chemokines, Il-8 and its receptors CXCR1/2 have been shown to be upregulated in several animal models following nerve injury and implicated in the development and maintenance of neuropathic pain and of the inflammatory hypernociception [21–22].

Interestingly, in a recent controlled study in patients with painful peripheral neuropathy, IL-6 and IL-8 gene expression were found significantly up-regulated in the affected skin biopsies [23] prompting a strong interest on these mediators as potential pharmacological targets for pain management.

The chemokine effects on pain sensation are complex and additional effort is required to clarify the role of these mediators in cancer derived algesia; only the identification of potent and selective chemokine clinical grade inhibitors will allow to fully understand the therapeutic potential of this approach.

Reparixin is an investigational, non-competitive allosteric inhibitor of the IL-8 receptors CXCR1 and CXCR2 [24] and it has been investigated as a potential novel antineoplastic agent in synergy with standard chemotherapy targeting the CXCR1^+^ breast cancer stem population [25–27]. In a recently completed phase Ib trial in metastatic breast cancer, the safety, pharmacokinetics and antitumor activity of reparixin in combination with weekly paclitaxel were investigated. In this study we observed a sizeable response rate with a low incidence and severity of peripheral neuropathy, suggesting that neurological toxicity, but not antitumor activity, may be reduced by reparixin administration [26].

In this paper we put to test for the first time the hypothesis that inhibition of IL-8 signaling could attenuate the development of chemotherapy induced neurotoxicity.

On these bases, this work aimed to clarify the effect of paclitaxel on CXCR1-CXCR2 dependent pathways in neuropathic pain and to elucidate the effect of a known CXCR1-CXCR2 inhibitor, reparixin, in counteracting neuropathic pain behavior and biomarkers in the CIPN context.

## RESULTS

### *In vivo* model: effect of reparixin in paclitaxel-induced mechanical and cold allodynia

**Figure 1:**
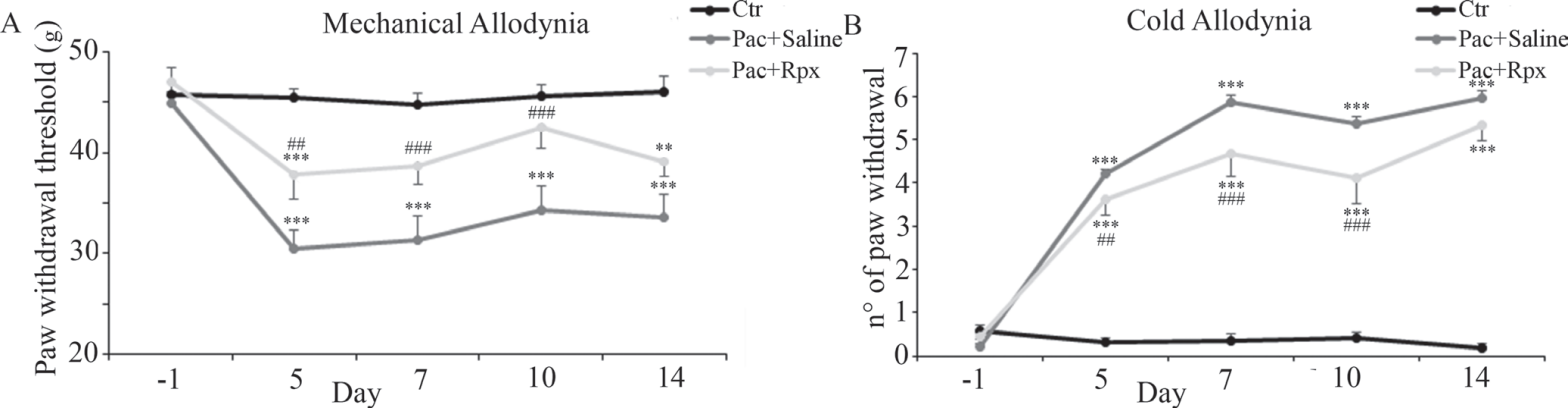
Effect of reparixin on paclitaxel-induced mechanical and cold allodynia: In control animals administered with paclitaxel vehicle i.p (Ctr, black dots) the paw withdrawal responses remained unchanged during the whole experimental period (panel **A** and **B**). Rats treated with paclitaxel + pumps filled with saline (Pac+Saline, gray dots), showed marked changes in paw withdrawal responses (panel A and B). Continuous infusion of reparixin (Pac+Rpx, light gray dots) was able to reduce in a significant way, paclitaxel-evoked mechanical (panel A) and cold (panel B) allodynia at days 5, 7 and 10, whereas no activity was determined on day 14, three days after the end of drug delivery. Data are shown as mean ± SE of 10 animals per group. ***P < 0.001, and **P < 0.01 vs the respective Ctr group; ^###^*P* < 0.001 and ^##^*P* < 0.01 vs the respective Pac+Saline group.

In control animals administered with paclitaxel vehicle i.p., the paw withdrawal threshold remained unchanged during the whole experimental period, with a trend corresponding to that achieved in naïve animals with received no treatment (data not shown). In contrast, paclitaxel-saline -treated animals showed marked changes in paw withdrawal responses, which are prominently displayed at 5 days after the first paclitaxel treatment (the first time point observation after paclitaxel injection) and persisted at least 2 weeks (Figure 1A).

In particular, in DPA test, paw withdrawal threshold of paclitaxel-saline-treated animals resulted significantly reduced at day 5, 7, 10 and 14 (Figure 1A). Similarly, in cold allodynia experiments, no paw withdrawal response was induced by acetone in control animals, indicating that acetone-evoked cold stimulation is not noxious in non neurophatic rats. On the other hand, the numbers of paw withdrawal threshold resulted significantly increased at days 5, 7, 10 and 14, in paclitaxel-saline-treated animals (Figure 1B).

Reparixin antinociceptive effect was visible over the time-course corresponding to drug delivery by micro-osmotic pump; conversely, the activity disappeared after the end of drug delivery by pump (day 11), indicating that the observed protection is strictly correlated to drug administration and action.

In fact, continuous infusion of reparixin (8mg/hr/kg) was able to reduce in a significant way, paclitaxel-evoked mechanical allodynia at days 5 (*P* < 0.01), 7 (*P* < 0.001), and 10 (*P* < 0.001), whereas no activity was determined at day 14, three days after the end of drug delivery (Figure 1A).

Likewise, animals treated with reparixin showed a significant reduction of cold allodynia at days 5 (*P* < 0.01), 7 (*P* < 0.001), and 10 (*P* < 0.001), whereas, no activity was determined at day 14, three days after the end of drug delivery (Figure 1B).

### *In vitro* models

**Figure 2:**
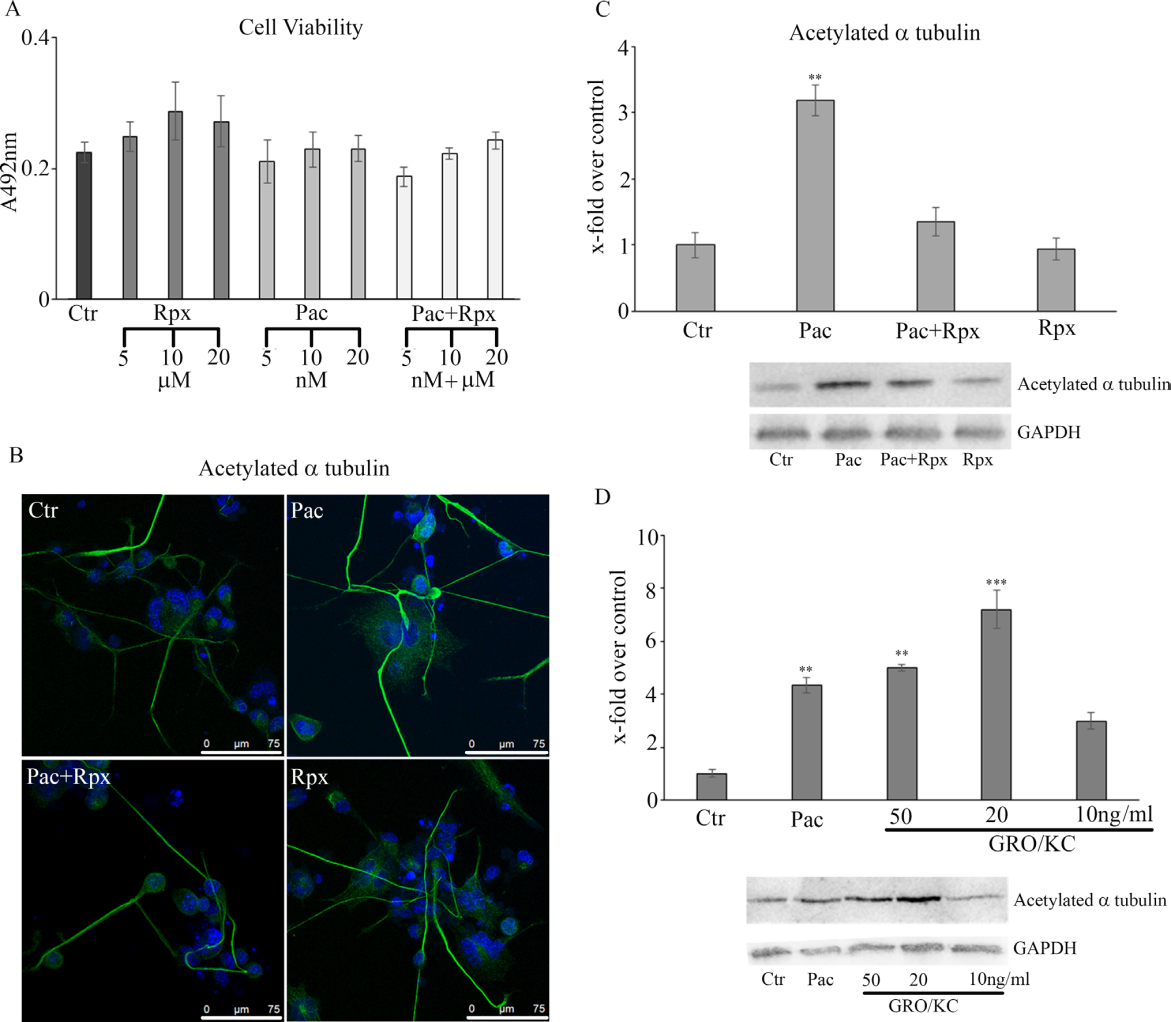
cell viability assay under the different treatments (**A)** Control, paclitaxel and reparixin treated neurons probed for anti-acetylated α-tubulin are shown (**B**) In panel (**C)** the western blotting and densitometric analyses for acetylated α-tubulin are reported. In order to determine whether the observed effects may be triggered by the CXCR2 natural ligand, GRO/KC, was used. In panel, (**D**) acetylated α-tubulin was re-assayed by western blotting under GRO/KC treatment at different concentrations, and compared to paclitaxel treatment. Data are mean ± SE of 3 different experiments. **p < 0.005; ***p < 0.0005. Bar = 75 μm.

The viability assay under the different treatments (Figure 2A) shows that paclitaxel, in the concentration-range of 5–20 nM, and reparixin, in the concentration range of 5–20 μM were ineffective in modulating cells viability.

For all the other experiments, reparixin was used at the final concentration of 10 μM and paclitaxel at the final concentration of 10 nM.

In Figure 2B, control (not treated), paclitaxel and reparixin treated neurons probed for anti-acetylated α-tubulin (markers of stable microtubules) are shown. In control cells, acetylated α-tubulin appears moderately present; a similar situation is found in cells treated with reparixin. In paclitaxel-treated neurons, in agreement with the literature [28], increased acetylated α-tubulin is observed. In fact, the fluorescence intensity in neurites appears more intense and an increase of neurite diameter and cytoskeleton organization is apparent. Acetylated α-tubulin in paclitaxel+reparixin-treated neurons appears similar to that of control conditions, thus indicating that reparixin presence counteracts paclitaxel effects.

Acetylated α-tubulin was assayed also by western blotting analysis. In Figure 2C, the western blotting and densitometric analyses for this marker are reported. In agreement with the morphological data, paclitaxel determines a significant increase of acetylated α-tubulin, while under combined treatments the protein appears at the same level of control cells. In order to determine whether the observed effects may be triggered by the CXCR2 natural ligand, GRO/KC, which is thought to play a role in the rat similar to that played by IL-8 in humans, was used. Acetylated α-tubulin was re-assayed by western blotting under GRO/KC treatment at different concentrations (Figure 2D), and compared to paclitaxel treatment. It is possible to observe that GRO/KC is able to increase acetylated α-tubulin at each concentration used.

**Figure 3:**
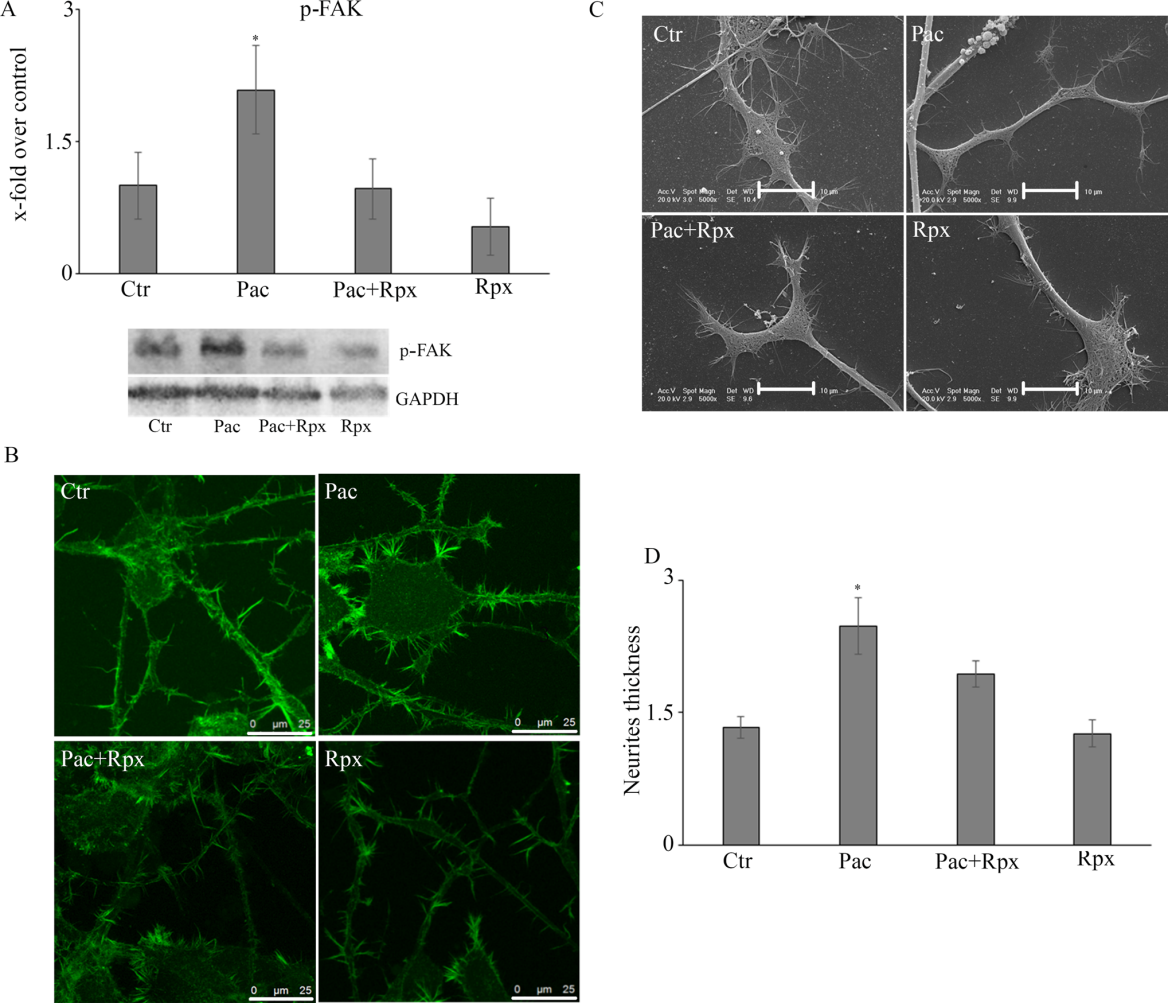
(**A**) Western blotting analysis for the active form of the protein of fokal adhesion, p-FAK. In panel (**B**) the localization for the cytoskeletal component actin on control and treated cells is reported. The SEM analysis were also performed and reported in panel (**C)**. By the mean of actin localization also neurite thickness was calculated (panel **D**). Data are mean ± SE of 3 different experiments. *p < 0.05; Bar = 25 μm.

The subsequent experiments were performed in order to dissect the signal transduction pathways, under GRO/KC control, eventually modulated by paclitaxel and/or reparixin (Figure 3). To this purpose the first enzyme studied was the active form of the protein of fokal adhesion, p-FAK. The protein is significantly increased by paclitaxel treatment, while under combined treatments it appears at the same level of control cells (Figure 3A).

In control and treated cells, the localization for the cytoskeletal component actin (Figure 3B) and the SEM analysis (Figure 3C) were also performed. By the mean of actin localization also neurite thickness (Figure 3D) was calculated. It is possible to observe that under paclitaxel treatment, neurites appear with a greater thickness than control cells. Under combined treatment cells appear as control ones.

**Figure 4:**
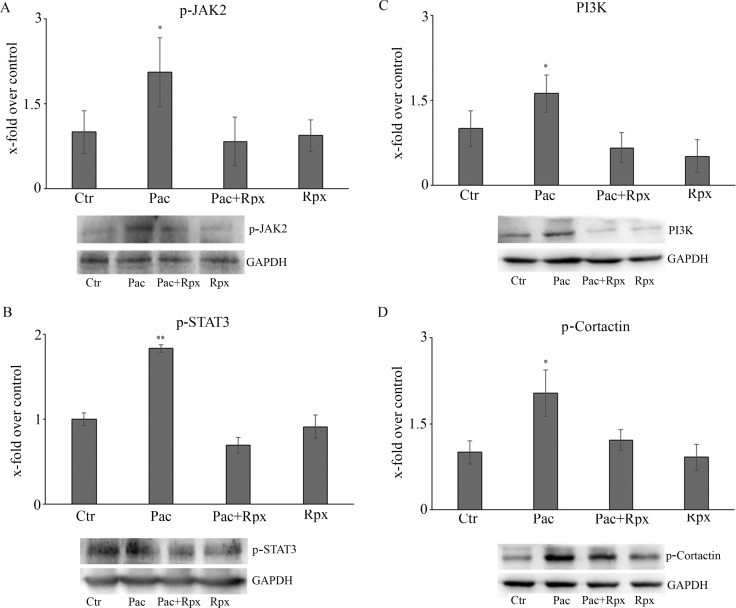
Western blotting analysis for the active form of the JAK2 protein, involved in the p-STAT3 signaling (**A**). In panel **B**, p-STAT3 levels, under the different conditions. In panels (**C** and **D)**, the pathway PI3K-p-cortactin is reported. Data are mean ± SE of 3 different experiments. *p < 0.05; **p < 0.005.

The other enzyme of CXCR1/CXCR2 pathway studied was the active form of the JAK2 protein, involved in the p-STAT3 signaling (Figure 4A), which in turn is involved in neuropathic pain and synaptic plasticity [29–30]. It is possible to observe that paclitaxel increases p-JAK2, while the presence of reparixin restores the control conditions. In the same Figure 4B, p-STAT3 levels, under the different conditions, are reported. Paclitaxel increases p-STAT3 levels, with respect to control, while reparixin counteracts this effect.

Finally, PI3K-p-cortactin pathway, which is involved in axonal arborization and synaptic plasticity, appears strongly up-regulated by paclitaxel, while the presence of reparixin counteracts also this effect (Figure 4C and 4D).

**Figure 5:**
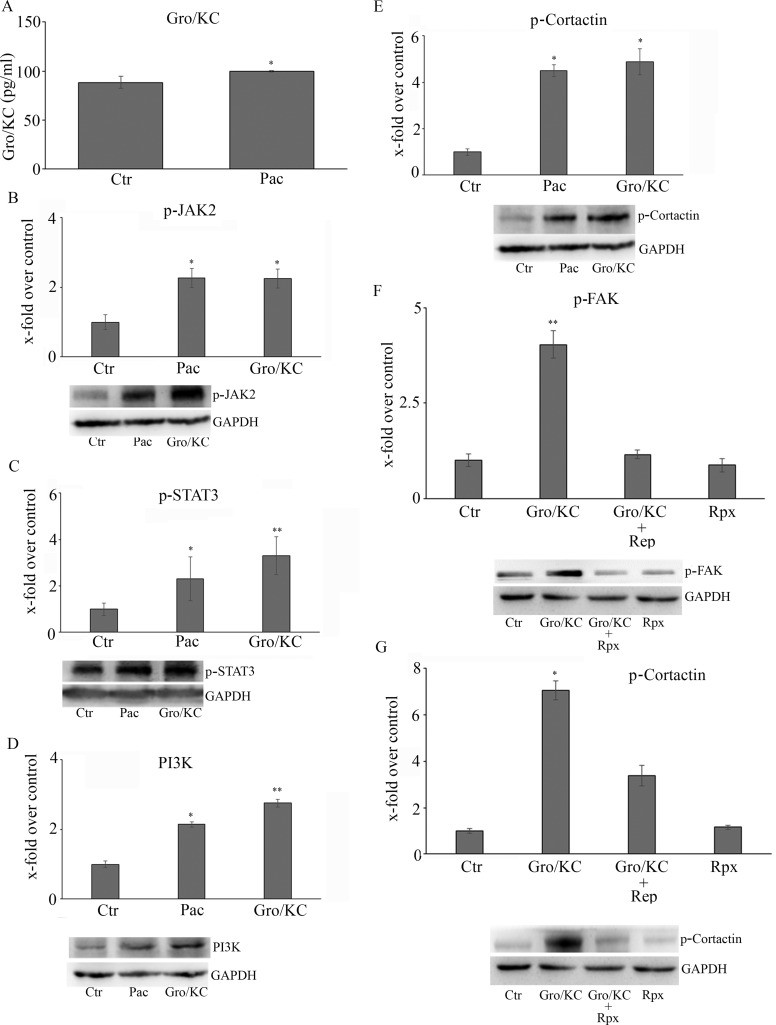
(**A**) GRO/KC 5 levels released into the *culture medium*, quantitated by ELISA assay The whole CXCR1/CXCR2-mediated pathway re-assayed in the presence of GRO/KC in the place of paclitaxel is shown in Panels (**B**–**E)**. The effects of reparixin on GRO/KC-induced p-cortactin and p-Fak is reported in Panels (**F** and **G)**. Data are mean ± SE of 3 different experiments. *p < 0.05; **p < 0.005.

With the aim to clarify the correlation between paclitaxel effects and GRO/KC effects, F11 cells were incubated with paclitaxel for 24h and levels of GRO/KC released into the culture medium were quantitated by ELISA assay. The obtained results showed that paclitaxel was able to induce a significant increase in GRO/KC release (Figure 5).

The whole CXCR1/CXCR2-mediated pathway was re-assayed in the presence of GRO/KC in place of paclitaxel, obtaining the same effects of paclitaxel (Figure 5), thus indicating that all the effects of taxol in inducing neuropathic pain biomarkers are under GRO/KC control.

Finally, the effect of reparixin on GRO/KC-induced p-cortactin and p-Fak was tested; in line with the previous experimental data, reparixin markedly inhibited both the biomarkers (Figure 5F and 5G).

## DISCUSSION

CIPN is a common and potentially severe adverse effect associated with MTSA therapy [31] and taxane-induced neuropathy is by far the most extensively investigated in preclinical and clinical studies. Such a neuropathy, usually presenting as sensory neuropathy, is more frequently associated with paclitaxel than docetaxel and seems to depend on the dose per treatment cycle, the schedule of treatment, and the duration of the infusion. Despite clinical efforts to minimize paclitaxel-induced neurotoxicity, no drugs are approved as of today to prevent or treat CIPN, which often requires dose reduction or treatment interruption with a major impact on patients quality of life and clinical outcome [4, 2, 5]. Taxanes bind the inner surface along the entire length of microtubules promoting their stabilization by suppression of microtubule shortening events [32]. In addition to their important function in cell division, microtubules play an essential role in cell morphology and intracellular transport [33]. Accordingly taxane-induced CIPN has been for a long time interpreted as a consequence of MTSA interference with normal microtubule-dependent axonal transport leading to interruption of axoplasmic flow and, as a consequence, to damage of peripheral nerves [34].

Along the last decades, great effort has been made to further investigate the underlying physiopathological mechanisms and additional evidences emerged [9, 35–38] suggesting several alternative mechanisms [6, 39]. Among those, extensive research has been dedicated to chemotherapy-induced inflammation as the potential common driver of CIPN. Several independent studies concur to demonstrate a chemotherapy-induced increase of peripheral pro-inflammatory and chemoactractant cytokine levels and an apparent correlation with behavioral toxicities and peripheral neuropathy [11, 40].

Inflammatory cytokines and chemokines (TNFα, IL-1β, IL-6, CCL2, IL-8), produced and secreted by neurons and by infiltrated macrophages in response to the chemotherapy-induced injury, can cause neurotoxicity not only by triggering the inflammatory response but also by modulating spontaneous nociceptor sensitivity and activity [41–42]. The key role of pro-inflammatory mediators in the development of CIPN has found important confirmations in preclinical studies that proved the efficacy of specific IL-1β and TNFα blockage in attenuating peripheral neuropathy in relevant animal models [16, 28, 43].

Chemokine signaling in leukocytes is mediated by the specific interaction with membrane receptors that belong to the large family of G protein-coupled receptors (GPCRs) [44]. Compelling evidence demonstrated that chemokine receptors are also expressed in neurons and astrocytes and significantly up-regulated during both early-phase and late-phase of chronic pain suggesting an implication of this pathway in neuroinflammation [45].

Among the chemokines family, IL-8 and its receptors CXCR1/2 have been emerging as one of the most promising target for the pharmacological management of CIPN due to its implication in sympathetic components of inflammatory hypernociception and in neuropathic nociceptive response [46–47]. Interestingly, a recent controlled clinical study showed a significant upregulation of IL8 gene expression in the skin biopsies of patients affected by CIPN [23]. Also, the results of a recently completed phase Ib trial in metastatic breast cancer, showed that combination of reparixin with weekly paclitaxel resulted in a sizeable response rate with a low incidence and severity of peripheral neuropathy, suggesting that taxane-induced neurological toxicity, but not antitumor activity, may be reduced by CXCR1/2 inhibition [27].

Starting from this observation and with the aim to dissect molecular mechanisms underlying the effects of CXCR1/CXCR2 blocking compounds in counteracting initiation and progression of paclitaxel-induced peripheral neuropathy, we evaluated direct effects of IL-8-induced signaling in DRG derived neurons cultures.

Tubulin acetylation participates the complex process regulating microtubule dynamics that is influenced by a number of different microtubule-associated proteins (MAPs), including the end binding proteins EB1 and EB3 implicated in the promotion of microtubule growth and axonal transport and in linking dynamic microtubules with the actin cytoskeleton [48–50]. In our experimental conditions, the paclitaxel-induced increase of α-acetylated tubulin in DRG derived neurons was efficiently counteracted by the presence of reparixin. Reparixin effects were also apparent on the signal transduction pathways dissected in the study. IL-8 is significantly up-regulated by paclitaxel in DRG derived neurons. Even though the observed expression increase was relatively modest, it was sufficient to trigger the activation of IL-8 signal upon paclitaxel treatment.

Under paclitaxel treatment DRG derived neurons were shown to express higher levels of p-JAK2 which in turn activates p-STAT3, known to be involved in neuropathic pain and synaptic plasticity [29–30]. Additionally, exposure to paclitaxel was shown to increase the active form of fokal adhesion kinase (p-FAK), involved in microtubule stabilization [51], and to activate the PI3K-p-cortactin pathway that ultimately leads to terminal axonal arborization and synaptic plasticity [52]. With the aim to confirm the hypothesis that paclitaxel-induced neurotoxic effects are mainly mediated by IL8, the expression of CXCR2 and the effects of GRO/KC treatment on F11 were investigated. CXCR2 was found expressed on F11 (data not shown) and receptor binding of IL8 triggered the same pathways activated by paclitaxel. In agreement with the observed *in vivo* effect, CXCR2 inhibition by reparixin significantly counteracted p-STAT3, p-FAK and PI3K-p-cortactin activation induced either by paclitaxel or GRO/KC.

In summary, the majority of the effects of paclitaxel on DRG derived neurons in culture seem to be mediated by the GRO/KC -dependent pathway and blockade of GRO/KC receptors by reparixin effectively inhibited *in vitro* and *in vivo* paclitaxel-induced neurotoxic effects.

In breast cancer models, reparixin showed the ability to reduce the CSC population *in vitro* and in tumor xenografts. It is remarkable how the signaling pattern activated by paclitaxel/IL8 in F11, putatively responsible for neurons sensitization, is largely the same pattern that in breast cancer tumor cells was reported to promote tumor growth and invasiveness. In a recent paper the effects of reparixin on paclitaxel-induced signaling was studied in highly purified mammospheres derived from triple negative MDA-MB231 breast cancer cells showing that its effect in breast cancer cells was mediated by inhibition of paclitaxel/GRO/KC-induced p-FAK, p-AKT, p-STAT3 and β-catenin activation [26].

FAK is a cytoplasmic tyrosine kinase identified as a key mediator of intracellular signaling by integrins involved in the regulation of different cellular functions in a variety of cells [53–54]. Consistent with reparixin pharmacological effects, p-FAK activation in breast cancer cells was previously reported to be implicated in anchorage-independent growth [55] and in breast cancer tumorigenesis and progression [56–60] whereas in sensory neurons β1-integrin activity and FAK phosphorylation at tyrosine 397 (FAKpY397) are linked to neuronal polarization as well as neurite outgrowth and branching [61].

The avoidance of pFAK activation by CXCR1/2 inhibition in breast cancer cells results in sensitization and reduction of migrative potential of stem cells, while the inhibition of the same pathway in peripheral sensory neurons, promoted by taxane treatment via exocrine and endocrine IL-8 production, may efficiently prevent microtubule acetylation, terminal axons arborization and synaptic plasticity thus modulating the key features of chemotherapy-associated neurotoxicity.

Since the cytotoxic effect of taxanes on cancer cells is strictly associated with the ability to stabilize microtubules and to promote net microtubule polymerization, the effect of reparixin treatment on α-tubulin association observed in sensory neurons was investigated with the aim to understand potential implication on cancer cells biology.

Biochemically, also in cancer cells paclitaxel induces α-tubulin expression and acetylation. While tubulin acetylation has historically been viewed as a marker but not a cause of microtubule stability, recent work suggests an active role of the tubulin acetylation/deacetylation process in the regulation of cell motility [62–64]. In particular, the inhibition of tubulin deacetylation mediated by two identified tubulin deacetylases HDAC6 and SIRT2 enhances sensitivity to the anti-motility activity of paclitaxel theoretically potentiating the anti-tumor effect of the drug [65–67]. In this perspective, we investigated in this paper the effect of paclitaxel on α-tubulin acetylation, the outcome of reparixin inhibition on paclitaxel-induced tubulin acetylation in MDA-MB231-derived breast cancer stem tumorspheres. The evidence that reparixin did not inhibit paclitaxel-induced tubulin acetylation in MDA-MB231 is coherent with the reported synergic effect of reparixin and paclitaxel on breast cancer cell survival, motility and invasiveness. The apparently divergent effect of reparixin combination on neural and cancer cells functionality is coherent with the specific pathways activated by CXCR1/2 receptors in the two different cellular contexts.

Overall the results described in this paper for the first time provide evidences that IL-8 and its receptors CXCR1/2 play a key role in the induction and progression of peripheral neuropathy and reinforce the hypothesis that CXCR1/2 inhibitors combined with standard taxane therapy might potentiate the taxane anti-tumor activity and reduce the chemotherapy-induced neurotoxicity. Since JAK2, STAT3 and PI3K pathways are also involved in several other activities such as apoptosis and senescence, that are two phenomena that could be induced by anticancer drugs, future investigations will be performed in order to evaluate if treatment with reparixin may have any effect on these phenomena as already described by other Authors in different cancer cells [68–70].

## MATERIALS AND METHODS

### *In vivo* model

#### Animals

The behavioral experiments were performed on male Wistar rats (200-250 g, Harlan Italy) housed in the animal care facility of the Department of Pharmacy of the University of Naples Federico II, Italy. Animals were housed in a room with controlled temperature (22±1°C), humidity (60 ± 10%) and light (12 h per day); food and water were available *ad libitum*. All behavioral tests were performed between 9:00 AM and 5:00 PM, and the animals were used only once. Animal care and manipulations were conducted in conformity with International and National law and policies (EU Directive 2010/63/EU for animal experiments, ARRIVE guidelines and the Basel declaration including the 3R concept). The procedure reported here were approved by the Institutional Committee on the Ethics of Animal Experiments (CVS) of the University of Naples Federico II and by Ministero della Salute under protocol n. 2014-00884607. Ten animals per group were used.

### Drug treatment

Reparixin L-lysine salt was administered by continuous infusion at the effective dose of 8mg/hr/kg [71] using micro-osmotic pumps (ALZET Model 2ML2 Charles River). Alzet model 2ML2 pump has an approximate 2ml reservoir that delivers a preloaded drug or vehicle solutions continuously for 14 days at a rate of 5 μl/h. Pumps were filled with reparixin L-lysine salt dissolved in sterile saline at a concentration of 375 mg/ml according to the instruction of the supplier. Briefly, pumps were filled with 2 ml of reparixin solution or vehicle (saline) using sterile syringe. Finally, pumps were kept in water bath overnight in stove at 37°C.

### Surgical implantation of osmotic pumps

Osmotic pumps were implanted under intraperitoneal (i.p.) anesthesia (100 mg/kg ketamine and 10 mg/kg xylazine). The osmotic pump was inserted through a surgical incision made on the back. Briefly, a small incision was made in the skin between the scapulae and a pocket was formed by spreading apart the subcutaneous connective tissues. The pump was inserted into the pocket with the flow moderator pointing away from the incision. Finally the incision was sutured and closed with sutures. Pumps were implanted 3 days before paclitaxel injection (day -3). The cessation of drug delivery was expected at day +11, as represented in the below scheme, since the duration of administration of 2ML2 mini pumps is 2 weeks.

**Figure:**
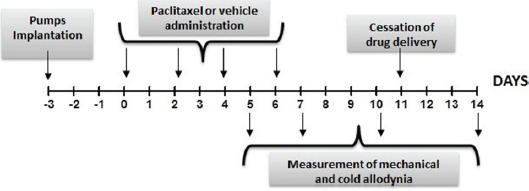


### Induction of neuropathy by paclitaxel

Rats received four once daily i.p. injections of paclitaxel (2 mg/kg/day; cumulative dose of 8 mg/kg; Tocris, Italy) or vehicle (15% PEG400, 5% Tween80 and saline, 1ml/kg/day), administered on alternate days (day 0, 2, 4, and 6) as described [9]. Behavioral testing was performed prior to paclitaxel/vehicle administration (day -1) in order to determine the basal values of the mechanical and cold nociceptive thresholds, and again on day 5-7-10-14 following paclitaxel/vehicle injection. as shown in the scheme above.

### Mechanical allodynia

To assess for changes in sensation or in the development of mechanical allodynia, sensitivity to tactile stimulation was measured using the Dynamic Plantar Aesthesiometer (DPA, Ugo Basile, Italy), which is an automated version of the von Frey hair assessment [72]. Individual animals were placed in Plexiglas boxes (30× 30× 25 cm) with a mesh metal floor covered by a plastic dome that enabled the animal to walk freely, but not to jump. When a trial is initiated, the device raises the filament to touch the foot and progressively increases force until the animal withdraws its foot, or until it reaches a maximum of 50 g of force (cut-off). The DPA automatically records the force at which the foot is withdrawn. This test does not require any special pre-training, just an acclimation period to the environment and testing procedure. Each paw was tested twice per session and the test was performed on both paws on day before (day -1) paclitaxel or vehicle first administration and then on 5th, 7th, 10th and 14th days after paclitaxel or vehicle first administration. No consistent left and right differences were observed. The means of the paws withdrawal (expressed in gram) were calculated from an average of four separate measures.

### Cold allodynia

Cold sensitivity was measured as the number of foot withdrawal responses after application of acetone to the dorsal surface of the paw [9, 73]. Individual animals were placed in Plexiglas boxes (30× 30× 25 cm). A drop of acetone (25 μL) was applied to the dorsal surface of paws with a syringe connected to a thin polyethylene tube while the rats was standing on a metal mesh. A brisk foot withdrawal response, after the spread of acetone over the dorsal surface of the paw, was considered as a sign of cold allodynia. The procedure was repeated three times at 5 minutes intervals on both paws. The mean of the paw withdrawal (expressed in number) was determined from an average of six separate measures. Cold responses were measured before paclitaxel or vehicle first administration (day -1) and then on 5th, 7th, 10th and 14th days after. No consistent left and right differences were observed.

### *In vitro* models

#### Cell culture and treatments

The F11 hybridoma cells (ECACC 08062601), chosen as model of dorsal root ganglion (DRG) neurons [74–75] were cultivated in DMEM (Euroclone, MI, Italy) medium supplemented with 10% FBS (Sigma-Aldrich St. Louis, CO, USA), 1% penicillin/streptomycin (Euroclone) and 1% glutamine (Euroclone) at 37°C, in a humidified 95% air-5% CO_2_ atmosphere. For all the experiments cells were used at 18th passage. For immunofluorescence analysis cells were seeded on coverslips at 1 × 10^4^ cells/cm^2^ for 24h. After, cells were differentiated with mouse NGF (mNGF) (from Dompé farmaceutici S.p.A., L'Aquila, Italy). mNGF was dissolved in DMEM with 1% penicillin/streptomycin and 1% glutamine (FBS free) at the final concentration 50 ng/ml. Medium was replaced every 3 days until complete differentiation, that happened after 7 days.

Following neuronal differentiation, neurons were treated for 24 hours with reparixin (Dompé farmaceutici S.p.A.; 10 μM final concentration), paclitaxel (Sigma-Aldrich; 10 nM final concentration) and the combination of the two molecules.

Reparixin stock solution (53mM) was prepared freshly dissolving 7,5 mg of reparixin in 0,5 ml of Phosphate Buffer in addition to 5 μl NaOH. Paclitaxel stocks solution (10 mM) was prepared dissolving the powder in DMSO and aliquots were stored at −20°C.

In some experiments, GRO/KC (PeproTech NJ, USA) was used (at 50, 20, 10 ng/ml) in comparison to paclitaxel. GRO/KC stock solution (0.1 mg/ml) was prepared dissolving the powder in 0.1% BSA in PBS; aliquots were stored at −20°C.

### MTS assay

Cell viability was determined at 24 h using Cell Titer One Solution Cell Proliferation Assay (Promega Corporation Madison, WI, USA) a colorimetric method based on 3-(4,5-dimethylthiazol-2-yl)-5-(3-carboxymethoxyphenil)-2-(4-sulfophenyl)-2H-tetrazolium (MTS). The quantity of formazan formed, as a function of viability, was measured at 492 nm using an ELISA plate reader, Infinite F200 (Tecan, Männedorf, Swiss). The assay was performed in quintuplicate. The results were expressed as absorbance at 492 nm.

### Immunofluorescence

Cells were fixed in 4% paraformaldehyde in PBS for 20 min at room temperature (RT) and permeabilized in methanol for 5 min at -20°C. Cells were then blocked with PBS containing 4% BSA for 30 min and incubated with the following primary antibodies diluted in the blocking solution overnight at 4°C: rabbit ß-tubulin (Abcam, Cambridge, UK ) 1:200; mouse α-tubulin (Abcam) 1:1000; mouse acetylated α-tubulin (Abcam) 1:500. Cells were then rinsed in PBS several times before incubation with secondary antibodies, goat anti rabbit conjugated with Alexafluor 633 (1:2000), goat anti mouse conjugated with Alexafluor 488 (1:2000), (Life Technologies, CA, USA) for 30 min at RT. After extensive washing, coverslips were mounted with Vectashield mounting medium with DAPI (Vector Laboratories Burlingame, CA, USA) and then observed at a Leica TCS SP5 confocal microscope (Mannheim, Germany).

### Phalloidin staining

Cells were fixed in 4% paraformaldehyde in PBS for 10 min at RT and permeabilized in TritonX100 0.1% for 5 min at −20°C. Cells were incubated overnight at 4°C with Phalloidin-FITCH (5mg/ml; Sigma-Aldrich) diluted in PBS. After extensive washing with PBS, coverslips were mounted with Vectashield mounting medium with DAPI (Vector Laboratories Burlingame, CA, USA) and then observed at confocal laser microscope.

### Neurite thickness

Neurite thickness was analyzed by ImageJ software by converting pixels related to single neurite in μm. For each neurite at least 3 different measurements at different levels were averaged. For each treatment at least 5 fields were counted.

### Western blotting

Control and treated cells were collected and lysated in ice-cold RIPA buffer (phosphate buffer saline pH 7.4 containing 0.5% sodium deoxycolate, 1% Nonidet P-40, 0.1% SDS, 5mM EDTA, 1% protease and phosphatase inhibitor cocktails, Sigma). Protein lysates (30 μg; 15 μg only for acetylated α-tubulin) were separated on 7–10% SDS–polyacrilamide gel and electroblotted onto polyvinyldifluoride membrane (PVDF; Sigma). Nonspecific binding sites were blocked by 5% non-fat dry milk (Bio-Rad Laboratories, Hercules, CA) in Tris buffered saline (TBS: 20mMTris– HCl, pH 7,4, containing 150 mM NaCl) for 30 min at RT. Membranes were then incubated overnight at 4°C with the following primary antibodies, diluted with TBS containing 0,1% Tween 20 (TBS-T) and 5% non-fat dry milk: mouse acetylated α-tubulin 1:1000 (Abcam), rabbit p-FAK 1:500 (Santa Cruz), rabbit p-JAK2 1:500 (Santa Cruz), rabbit PI3K 1:500 (Abcam), pCortactin (1:1000) (Abcam), rabbit GAPDH 1:5000 (Santa Cruz), goat pSTAT3 1:200 (Santa Cruz). As secondary antibodies, peroxidase conjugated anti-rabbit or anti mouse IgG (1:1000; Vector Laboratories, Burlingame, CA) and anti-goat (1:10000 Santa Cruz) were used. Immunoreactive bands were visualized by ECL (Pierce), according to the manufacturer's instructions. The relative densities of the immunoreactive bands were determinated and normalizated with respect to GAPDH, using ImageJ software. Values were given as relative units (RU).

### Scanning electron microscopy analysis

For Scanning Electron Microscopy (SEM) observations, cells were cultured at a density of 10.000 cells/cm^2^ on a round cover slip in a 4 well plate and treated with paclitaxel, reparixin or reparixin in combination with paclitaxel for 24 h. The morphology of the F11 cells was observed using scanning electron microscope (SEM Philips XL-30 CP). The cells were fixed with 3% glutaraldehyde in PBS at RT for 20 min and then washed three times in PBS. Subsequently, the cells were dehydrated in graded series of cold ethanol (30, 50, 70, 85, 90, 95 and 100%; 5 min, two times each). Dehydrated samples were dried further in a critical point dryer and coated with gold (10 nm) before analysis.

### Quantitation of GRO/KC

The quantification of GRO/KC released in the medium was performed using ELISA (enzyme-linked immunosorbent assay) kit (Thermo Fisher Scientific Waltham, MA, USA) using a microplate reader Infinite F200 (Tecan). Briefly, a target-specific antibody had been pre-coated in the wells of the supplied microplate, samples and standards were then added into these wells and bind to the immobilized (capture) antibody. The sandwich was formed by the addition of the second (detector) antibody, binding to the target on a different epitope from the capture antibody. A conjugated enzyme had been incorporated into the assay. After incubation periods and wash steps to remove unbound antibody from the plate, a substrate solution was added reacting with the enzyme-antibody-target complex to produce measurable signal, measured at 450 nm using an ELISA plate reader. The intensity of this signal was proportional to the concentration of target present in the sample and the results were expressed in pg/ml. All ELISA assays were performed in quintuplicate.

### Statistics

For the *in vivo* results, all data were presented as the mean ± SE. Analysis of data was conducted using GraphPad Prism (GraphPad Software Inc., San Diego, CA). The significance of differences between groups was determined by two-way analysis of variance (ANOVA) followed by Bonferroni post hoc tests for multiple comparisons. The level of significance was set at *P* < 0.05.

For the *in vitro* results, data were expressed as mean ± SE. Statistical analysis was performed by the analysis of variance (ANOVA) followed by Scheffe's post hoc test analysis. The level of significance was set at *P* < 0.05.

## SUPPLEMENTARY MATERIALS FIGURE


