# Supplementary material for: CXCR1/2 pathways in paclitaxel-induced neuropathic pain
Source: Oncotarget. 2017 Feb 20;8(14):23188–201. doi: 10.18632/oncotarget.15533 (PMC5410296; doi:10.18632/oncotarget.15533)
Supplement: Supplementary file 1 [file oncotarget-08-23188-s001.pdf]

# CXCR1/2 pathways in paclitaxel-induced neuropathic pain

## Supplementary Materials

### Experimental procedure

SHSY5Y human neuroblastoma cells (ATCC) were cultured in DMEM supplemented with N2 differentiation agent for obtaining differentiation.

To evaluate the expression of se CXCR1 and CXCR2, differentiated neuroblastoma cells were dissociated and the single-cell suspensions ($1 \times 10^6$ cell/tube) were kept, for 15 minutes at Room Temperature (RT), with 2% formaldehyde in PBS. Cells were washed with PBS and then incubated, for 1 hour at RT, with selected primary antibodies: monoclonal anti-CXCR1 (clone 42705,1:100, R&D Systems Inc., Minneapolis, USA), anti-CXCR2 (clone 48311, 1:100, R&D Systems Inc. (Minneapolis, USA), After washing with PBS, the cells were incubated for 1 hour at RT, with secondary AlexaFluor 488-conjugated anti-mouse or anti-rabbit IgG antibodies (Molecular Probes, Life Technology, Carlsbad, CA, USA) diluted 1:2000 in PBS containing 4% BSA. Cells were then washed with PBS and the population of interest was gating according to its Forward Scatter (FSC)/Side Scatter (SSC) criteria. 10,000 events were acquired for each sample and analyzed by CellQuest software (BD Biosciences). (Becton Dickinson Biosciences, BD, San Diego, CA).

For western blotting analysis for p-FAK the same procedure describerd in the ms was followed.

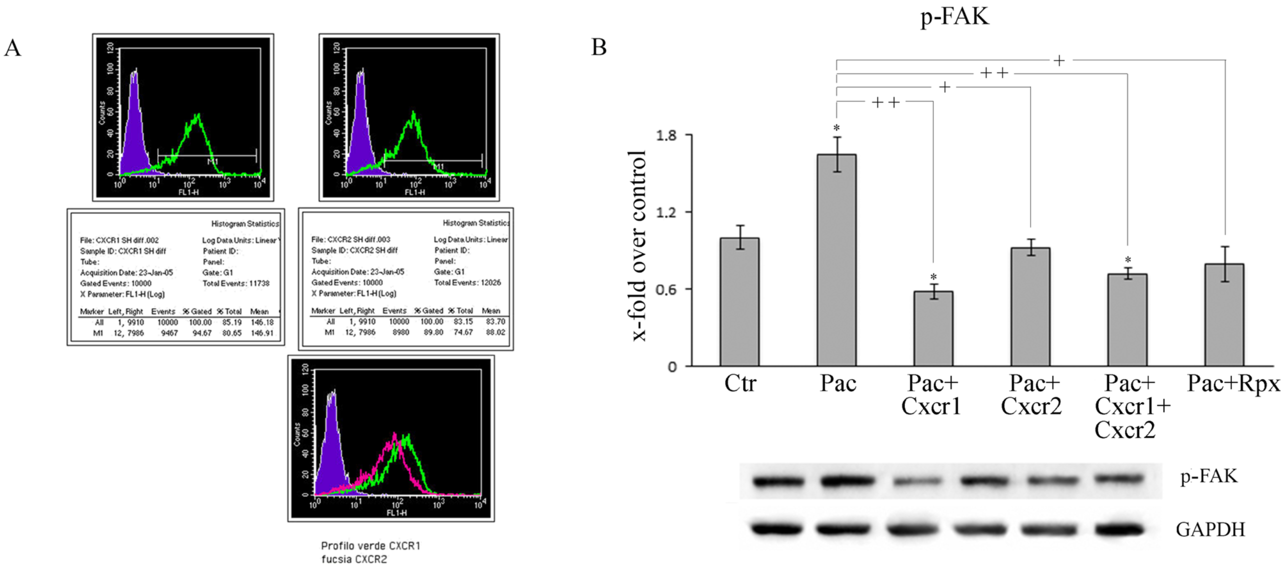


**Supplementary Figure 1: Cytofluorimetric analysis for CXCR1 and CXCR2 in differentiated neuroblastoma cells is reported on the right; on the left side, western blotting analysis for p-FAK in differenziated neuroblastoma cells treated with pac and/or with anti-CXCR1 or CXCR1 antibodies.** Data are mean ± ES of 3 different experiments. *$p < 0.05$ statistical analysi treated vs control; +, $p < 0.05$; ++, $p < 0.005$, statistical analysis combo treatment vs pac treatment.
